# Modification of Rigid Polyurethane Foams with Straw Additive: Influence of Chemical Treatment and Content on Performance Properties

**DOI:** 10.3390/polym17182440

**Published:** 2025-09-09

**Authors:** Anna Strąkowska, Justyna Miedzianowska-Masłowska, Sylwia Makowska

**Affiliations:** Institute of Polymer and Dye Technology, Faculty of Chemistry, Lodz University of Technology, 90-537 Lodz, Poland; justyna.miedzianowska@p.lodz.pl (J.M.-M.); sylwia.makowska@p.lodz.pl (S.M.)

**Keywords:** polyurethanes, rigid polyurethane foams, modification, natural fillers, cellulose, straw

## Abstract

This work aimed to synthesize rigid polyurethane foams with improved functional properties through modification with the addition of cellulose in the form of straw: unmodified, silanized, and silanized with the addition of fumed silica. The prepared rigid polyurethane foams contained 0.5; 1; and 3 parts by weight of the modifier about the weight of the polyol used. As part of the work, a number of tests were carried out to determine the impact of the modifiers used on the reaction kinetics and on the functional properties of rigid polyurethane foams. Silanization improved thermal stability and interfacial compatibility, while silica further enhanced porosity and surface activity. The optimal properties were obtained at low loadings: 0.5 wt.% provided the best mechanical strength, and 1 wt.% yielded the most uniform cell morphology and density. Higher contents increased porosity, reduced strength, and lowered water resistance. Dynamic mechanical analysis confirmed predominantly elastic behavior, with silica-modified fillers offering the most stable thermomechanical response. Overall, even small amounts of modified straw enhanced mechanical, structural, and water-resistant properties, demonstrating its potential as a sustainable and cost-effective biofiller for eco-friendly polyurethane foams.

## 1. Introduction

Polyurethane (PUR) foams are highly versatile polymeric materials used across numerous industries, including construction, automotive, furniture, aerospace, and packaging. Their widespread application stems from their unique combination of lightweight structure, excellent insulation properties, mechanical strength, and chemical resistance. PUR foams are produced through the reaction of polyols with isocyanates, forming a cross-linked polymer network that gives them their characteristic cellular structure. Depending on the formulation and processing conditions, PUR foams can be tailored to exhibit a range of properties suitable for different applications [1].

The growing interest in polyurethane foams stems from the simplicity of their manufacturing process and the wide range of properties that can be modified by altering the chemical structure of polyurethane or incorporating specific fillers [2].

Currently, recycled fillers are increasingly used to reduce production costs and decrease the consumption of petroleum-based raw materials, making polyurethane materials more environmentally friendly. Additionally, these fillers are readily available and can positively influence the functional properties of the resulting foams [3,4].

In recent years, there has also been a growing interest in using biofillers such as cellulose, lignin, flax, hemp, starch, sugarcane, and wood flour to reinforce rigid polyurethane foams. Studies indicate that these additives can increase the cellular density of foams and enhance their mechanical properties, including compressive strength [4,5]. However, the final properties of the foams depend not only on the type of biofiller used but also on its concentration. While improvements in certain functional properties are often observed, other parameters may deteriorate [5].

Cellulose is a biosynthetic product derived from plants, animals, and bacteria [6]. Its use as a natural filler can enhance the properties of polyurethane foams due to the inherent affinity between isocyanate groups (R-N=C=O) and the hydroxyl groups (-OH) of cellulose [7].

Nanocellulose refers to cellulose extracts or processed materials with defined structural dimensions at the nanoscale. The increasing demand for high-performance polyurethane materials with improved mechanical and physical properties makes nanocellulose one of the most promising renewable materials for advanced applications. Nanocellulose fillers possess unique characteristics, such as nanoscale dimensions (resulting in a high surface-to-volume ratio), non-toxicity, biodegradability, and biocompatibility [6,7].

Natural cellulose fibers are obtained from cereal plants, wood, flax, starch, and various other sources. They are lightweight, cost-effective, and biodegradable [8,9]. Polyurethane composites reinforced with cellulose fillers exhibit good mechanical properties and contribute to improved thermal insulation and electrical resistance. In recent years, straw has gained increasing attention as a filler, offering a sustainable alternative to conventional reinforcement materials in polyurethane composites [8,10].

One of the main challenges in utilizing natural fibers and biofillers in PUR foams is their hydrophilic nature, which reduces compatibility with the hydrophobic polymer matrix. This can lead to poor dispersion and weak interfacial adhesion, negatively impacting mechanical properties. To overcome this issue, various surface modification techniques are employed, including chemical treatments (acid or alkaline treatments, acetylation), oxidation and peroxidation processes, or silane coupling agents, which enhance the interaction between fiber and polymer, leading to stronger adhesion and better mechanical performance. Among these, silane treatment has proven particularly effective, as it modifies the fiber surface to improve bonding with polyurethane, resulting in foams with enhanced strength, flexibility, and thermal stability.

As the demand for lightweight, high-performance, and environmentally friendly materials continues to grow, polyurethane foams reinforced with biofillers will play an increasingly important role in various industries. Ongoing research aims to optimize the composition and processing of these foams to achieve the best balance between performance, sustainability, and cost-effectiveness. By integrating advanced bio-based additives and nanomaterials, future polyurethane foams can offer improved mechanical durability, fire resistance, and energy efficiency, contributing to a more sustainable materials industry.

Therefore, in this work, an attempt was made to assess the effect of using unmodified straw, silanized straw, and silanized straw with the addition of silica on the physicochemical and mechanical properties of rigid polyurethane foams.

The literature provides numerous examples of studies on the use of natural biofillers in polyurethane composites [11,12,13]. However, the use of chemically modified straw, especially in combination with mineral nanofillers, remains a relatively unexplored area, especially in polyurethane composites. The silanization process significantly improves the compatibility of fibers with the polyurethane matrix, while the introduction of an additional mineral phase, such as silica, can act synergistically, influencing the microstructure of foams, reducing moisture absorption, and enhancing the material’s mechanical and thermal properties. The research concept of this study, therefore, involves not only comparing the effects of using unmodified and modified straw as a filler, but also evaluating the innovative approach of combining biofillers with inorganic additives. This approach allows for the analysis of potential synergistic effects between components, thus enabling the creation of a new generation of insulating materials with improved performance parameters and increased durability.

The obtained results may contribute to the development of modern, ecological insulating materials with improved functional properties, while supporting the idea of sustainable development through the use of renewable raw materials and reducing the consumption of conventional petrochemical raw materials.

## 2. Materials and Methods

### 2.1. Materials

Polyurethane system:

The water-blown PUR foams used in this study were obtained from a two-component system supplied by Purinova Sp. z o. o. (Bydgoszcz, Poland), after mixing the polyol (Izopianol 30/10/C) and the diphenylmethane diisocyanate (Purocyn B). The polyol is a mixture of components containing polyester polyol (hydroxyl number ca. 230–250 mg KOH/g, functionality of 2), catalyst (N, N-Dimethylcyclohexylamine), flame retardant (Tris(2-chloro-1-methylethyl) phosphate), a chain extender (1,2-propanediol), and water as a blowing agent.

Straw additives:

Unmodified cereal straw (S)(wheat, rye, triticale) was obtained from local farms (Poland) and ground in a ball mill.

Straw silanized with 3-aminopropyl (diethoxy) methylsilane (Sigma-Aldrich, Poznan, Poland) (SS). The powdered straw was immersed in a 5% solution of silane in ethanol and placed in an ultrasonic cleaner UP400S from Hielscher Ultrasound Technology (Coatesville, PA 19320, USA) for 2 h. The solvent was then evaporated using a Laborota 4001 efficient rotary evaporator from Heidolph (Schwabach, Germany), and the residue was dried at 70 °C, obtaining a solid mass of silanized straw in powder form.

Straw silanized with the addition of pyrogenic silica Aerosil 380 (Evonik, Essen, Germany) (SS-SiO_2_) with a specific surface area of 380 g/m^2^. A powder containing 5 parts by weight of powdered silanized straw (obtained in the above step) to 1 part of silica was obtained using a mechanical process in which the initial powders were ground and simultaneously mixed using a Fritsch Pulverisette (Poznan, Poland) 5 ball mill for 2 h, with a break of 30 min after 1 h.

### 2.2. Methods and Instruments

The oil absorption value (DBPA) was determined using a Brabender Absorptometer C (Brabender, Duisburg, Germany). The method involved measuring the torque generated during the mixing of 20 g of filler with n-dibutyl phthalate (DBP), which was added at a constant rate of 4 mL/min. The DBPA value represents the amount of DBP absorbed per 100 g of filler at the point of maximum torque. The test was conducted following ASTM D2414.

Sieve analysis was performed using a vibratory shaker, a set of sieves with nominal mesh sizes of 0.25, 0.125, 0.063, and 0.045 mm, and a Radwag (Lodz, Poland) laboratory scale with an accuracy of 0.01 g. The analysis was conducted on straw samples weighing 20.00 g. The procedure was carried out using dry sieves at an amplitude of 1.5 mm for 3 min on a vibratory shaker.

Fourier transform infrared (FTIR) spectrum of fifiller was recorded using a Nicolet 6700 FTIR spectrometer and OMNIC 3.2 software (Thermo Scientifific, Waltham, MA, USA)

The thermal stability of the filler was evaluated using a Thermogravimetric Analyzer (TGA, Mettler Toledo, Greifensee, Switzerland). The analysis was performed under an argon atmosphere, with the temperature ranging from 25 to 700 °C and a heating rate of 10 °C/min.

The morphology of the filler was examined using scanning electron microscopy (SEM). Images were acquired with a LEO 1530 Gemini SEM (Zeiss, Oberkochen, Germany).

The process of obtaining rigid polyurethane foams included three stages. The first stage included latent time, which was measured from the moment the components were mixed until the reaction mixture began to increase in volume. The next stage was the growth time, which was measured from the moment the reaction mixture began to increase suddenly until it reached its maximum volume. The last stage concerned the gelation time (stabilization), which was measured from the moment the foam volume ended to the time when the surface viscosity of the foam disappeared.

The apparent density of the foams was determined following ISO 845 [14] by calculating the ratio of each sample’s mass to its volume. Measurements were taken from five samples per foam type, and the average value was reported.

Compressive strength (σ10%) was measured following ISO 844 [15]. Tests were conducted using a Zwick Z100 Testing Machine (Zwick/Roell Group, Wroclaw, Poland) equipped with a 2 kN load cell, operating at a crosshead speed of 2 mm/min. The compressive strength was determined based on the load required to produce 10% strain.

A minimum of five samples per series were tested, and the average values were calculated.

The three-point bending test was carried out according to ISO 178 [16] using the same Zwick Z100 Testing Machine. Samples were deflected at a rate of 2 mm/min, and at least five measurements were taken for each foam series. The average flexural stress at break was calculated from the individual results.

Short-term water absorption testing was conducted using the partial immersion method, which involved weighing each 40 × 40 × 40 mm^3^ cube sample, then partially immersing it in water to a depth of 10 mm and leaving it at room temperature for 24 h. After this time, the foam samples were weighed again to determine water absorption.

The thermomechanical properties of rigid polyurethane foams were investigated using dynamic mechanical analysis (DMA), which was performed on a TA Instruments (New Castle, DE, USA) ARES-G2 rotational rheometer using torsional geometry on pre-cut foam discs of 20 mm diameter and 2 mm thickness. Measurements were performed over a temperature range of 20 to 250 °C, using a vibration frequency of 1 Hz, a heating rate of 10 °C/min, and a strain of 0.1%.

## 3. Results and Discussion

### 3.1. Characteristics of Biofillers

#### 3.1.1. Oil Absorption and Torque Analysis of Fillers

**Figure 1:**
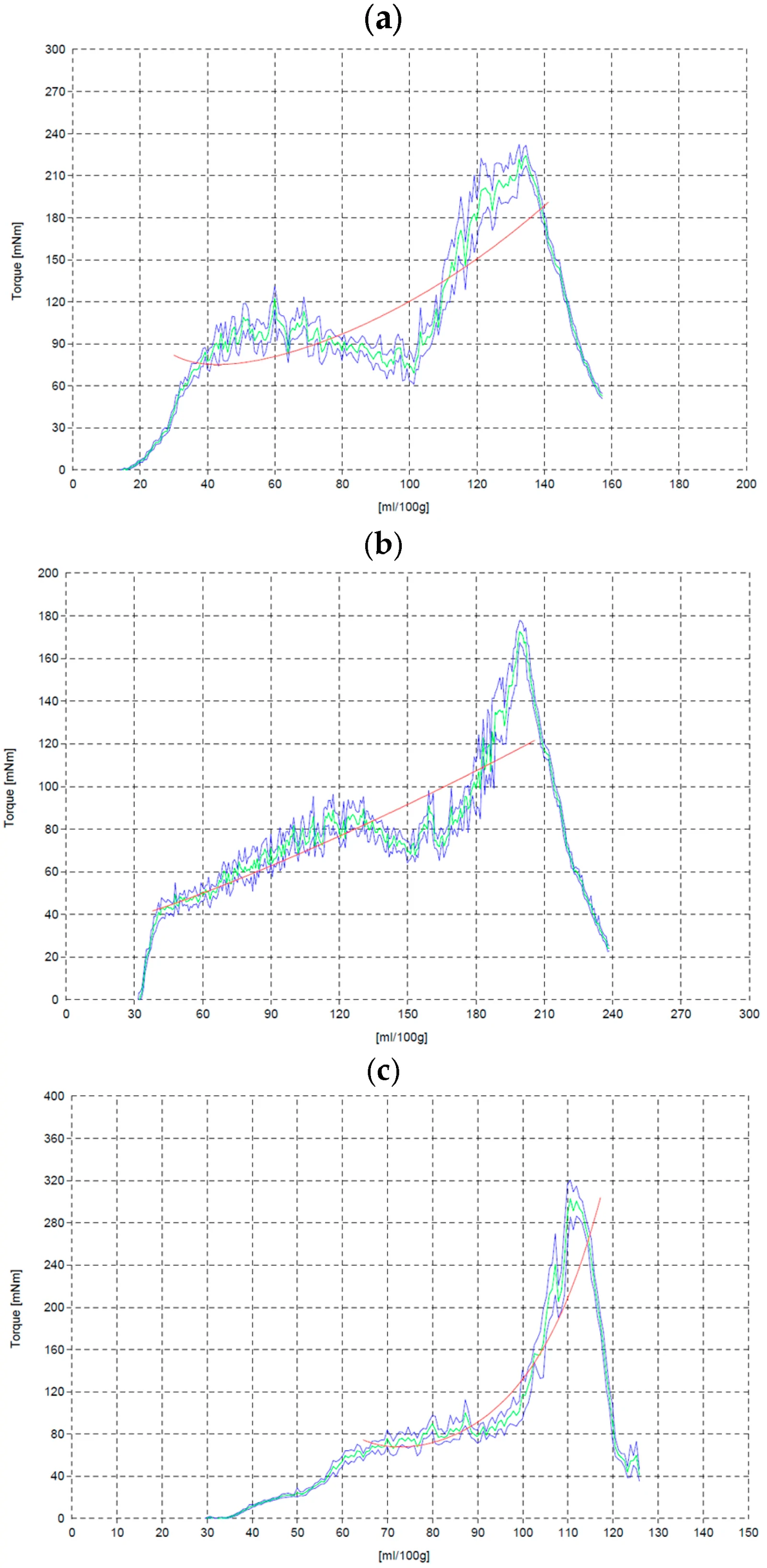
Torque as a function of the amount of dosed dibutyl phthalate (DBP) for (**a**) unmodified straw fillers, (**b**) silanized straw fillers, and (**c**) hybrid fillers composed of silanized straw and Aerosil 380.

The oil absorption number is an analytical method used to assess the structure and porosity of filler particles. During the measurement, dibutyl phthalate (DBP) is gradually added from a burette and penetrates the internal voids and surface pores of the filler structure. The higher the structural complexity and porosity of the filler, the greater the amount of DBP that is absorbed, reflected in a higher oil absorption number. Table 1 presents the oil absorption values and the maximum torque recorded during DBP dosing for the analyzed fillers. Subsequently, Figure 1 illustrates the dependence of torque on the amount of DBP dosed. As DBP penetrates into the pores and voids of the filler structure, the torque increases with the amount of DBP added until the filler’s absorption capacity is reached. The slope and final values of these curves therefore reflect the porosity and structural complexity of the individual fillers. In other words, each curve shows how a particular filler resists mixing during DBP penetration and how much DBP is absorbed until saturation, which directly corresponds to its oil absorption number and pore accessibility.

The highest oil absorption number was recorded for silanized straw, indicating that this filler exhibits the most developed porous structure and the greatest number of inter-aggregate voids. This promotes the formation of a self-assembled filler network within the polymer matrix. Such structural characteristics are critical for the reinforcing effect typically associated with active fillers. Therefore, bio-composites containing chemically modified straw are expected to demonstrate significantly improved mechanical properties.

It is also noteworthy to consider the maximum torque values measured during DBP dosing. The lowest resistance to mixing (122 mNm) was observed for the silanized straw, suggesting better dispersibility and reduced mechanical resistance during DBP penetration. In contrast, the unmodified straw showed a higher torque (191 mNm), indicating lower pore accessibility and increased resistance to DBPA.

The highest torque was recorded for the hybrid filler composed of silanized straw and Aerosil 380 (304 mNm), suggesting significant resistance during mixing. This behavior may result from the tendency of the filler particles to agglomerate, which hinders DBP diffusion and reduces the ability to form an extended three-dimensional structure within the foams, despite the theoretical high surface activity of the components.

The obtained results further confirm the beneficial impact of the silanization process on the performance characteristics of lignocellulosic bio-fillers and their reinforcing potential in polyurethane composites.

#### 3.1.2. Characterization of Particle Size Distribution in Fillers: A Sieve Analysis Approach

One of the fundamental methods for evaluating the particle size distribution of solid materials is sieve analysis, which involves the mechanical separation of grain fractions using a set of standardized sieves with defined mesh sizes. This method enables the determination of the granulometric composition of a material, i.e., the percentage content of individual particle size fractions relative to the total sample mass. This parameter is of critical importance when assessing the properties of particulate materials, as the particle size distribution significantly affects various functional properties, including mixability, surface reactivity, thermal conductivity, and mechanical performance. Ensuring uniformity in particle size is particularly important for achieving consistency in the properties of final products, which is especially relevant in the case of fillers used in polymer composites. The results are summarized in Table 2.

Based on the obtained particle size distributions, it can be concluded that the use of a ball mill resulted in highly effective comminution of all the analyzed fillers. For each filler, more than 75% of the particles were smaller than 0.125 mm, while the content of particles larger than 0.5 mm did not exceed 9%. For the unmodified straw filler, the most abundant fractions were particles within the 0.063–0.125 mm and <0.045 mm ranges, each accounting for approximately 30% of the total mass. In the case of silanized straw, the finest particles (<0.045 mm) were also the most dominant, comprising about 28.5%. However, this group exhibited a slightly higher proportion of particles larger than 0.25 mm (4.6%) compared to the unmodified straw, likely due to structural changes induced by the chemical modification process. Even finer comminution was achieved for the hybrid fillers. This effect was particularly pronounced for the filler composed of silanized straw and silica, for which particles smaller than 0.063 mm accounted for nearly 70% of the sample mass. The high degree of particle size reduction in this case is attributed to the additional grinding process using a planetary mill, which not only further reduced the straw particle size but also facilitated the mechanical deagglomeration of the silica clusters.

#### 3.1.3. FTIR Spectroscopic Analysis of Chemically Modified Straw-Based Fillers

**Figure 2:**
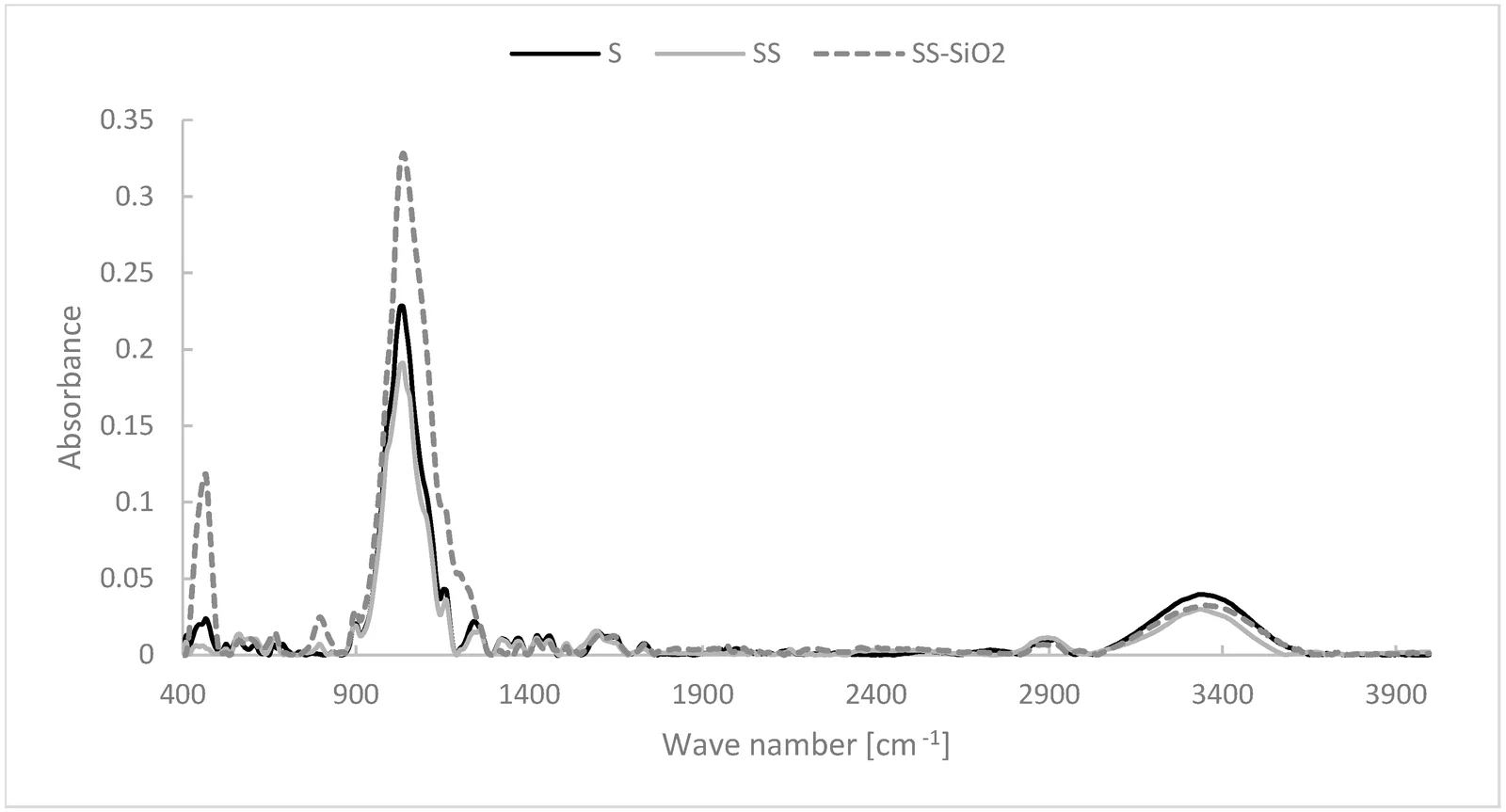
FTIR spectra of unmodified straw, silanized straw, and hybrid fillers.

Fourier-transform infrared (FTIR) spectroscopy was employed to investigate the structural changes in straw-based fillers resulting from chemical modification (silanization). The IR spectra of straw-based fillers are presented in Figure 2.

A broad absorption band observed in the 3000–3600 cm^−1^ range, with a maximum at approximately 3300 cm^−1^, corresponds to O–H stretching vibrations of hydroxyl groups present in cellulose, hemicellulose, and lignin. The reduced intensity of this band in the silanized straw indicates a decrease in hydrogen bonding, suggesting a reduction in the hydrophilic character of the filler surface due to the chemical modification [17]. The absorption bands in the 2950–2800 cm^−1^ range are attributed to symmetric and asymmetric C–H stretching vibrations of methyl and methylene groups. An increase in the intensity of these bands in the silanized straw may be associated with the introduction of additional alkyl groups during the silanization process [18]. The peak at 1730 cm^−1^ is assigned to the C=O stretching vibrations of acetyl groups in hemicellulose and lignin [19].

The absorption bands in the 2950–2800 cm^−1^ range are attributed to symmetric and asymmetric C–H stretching vibrations of methyl and methylene groups. An increase in the intensity of these bands in the silanized straw may be associated with the introduction of additional alkyl groups during the silanization process [18]. The peak at 1730 cm^−1^ is assigned to the C=O stretching vibrations of acetyl groups in hemicellulose and lignin [19]. A decrease in the intensity of this peak in the silanized straw suggests partial removal of these components from the filler surface. Peaks around 1650 and 1620 cm^-1^ correspond to C=O stretching vibrations of acetyl esters in hemicellulose and aldehyde groups in lignin, respectively. The diminished intensity of the 1650 cm^−1^ peak in the silanized straw indicates a reduction in hemicellulose content. The bands at approximately 1550 cm^−1^ and 1450 cm^−1^ are associated with C=C stretching vibrations in the aromatic rings of lignin [20]. The similar intensity of these bands in both unmodified and silanized straw suggests that the aromatic structure of lignin remains largely intact after silanization. Absorption bands at 1420 cm^−1^ and 1360 cm^−1^ are attributed to asymmetric and symmetric bending vibrations of C–H and –CH_3_ groups, respectively [21]. The 1360 cm^−1^ band also encompasses C–OH stretching vibrations in cellulose. The peak at 1230 cm^−1^ corresponds to C–O and C=O stretching vibrations in lignin [22]. A slight decrease in its intensity in the silanized straw may indicate partial removal of lignin from the filler surface. A prominent peak at 1030 cm^−1^ is characteristic of asymmetric C–O–C stretching vibrations in hemicellulose [23]. The reduced intensity of this peak in the silanized straw further supports the partial removal of hemicellulose due to the chemical modification.

FTIR spectroscopy was also utilized to compare the structural differences between silanized straw and hybrid fillers composed of silanized straw and Aerosil 380 silica. In the hybrid fillers, a slight increase in the intensity of the 3000–3600 cm^−1^ band was observed, which can be attributed to the presence of hydroxyl groups on the silica surface, indicating an increase in surface hydrophilicity. Enhanced intensity of bands around 1230 cm^−1^ and 850 cm^−1^ in the hybrid fillers may result from the formation of new C–O–Si bonds between silanol groups of the silica and C–O groups on the lignin surface during the mechanical modification process [24,25]. The strong absorption band at 1030 cm^−1^ in the hybrid fillers is due to overlapping Si–O–Si stretching vibrations characteristic of silica and Si–O–C vibrations [26], indicating successful integration of silica into the filler structure. Additionally, peaks around 470 cm^−1^ correspond to Si–O bending vibrations [27].

The similarity of the IR spectra of the hybrid fillers to that of the silanized straw suggests that the mechanical modifications did not adversely affect the filler structure, resulting in homogeneous hybrid materials.

#### 3.1.4. Thermogravimetric Characterization of Straw-Based Biofillers

**Figure 3:**
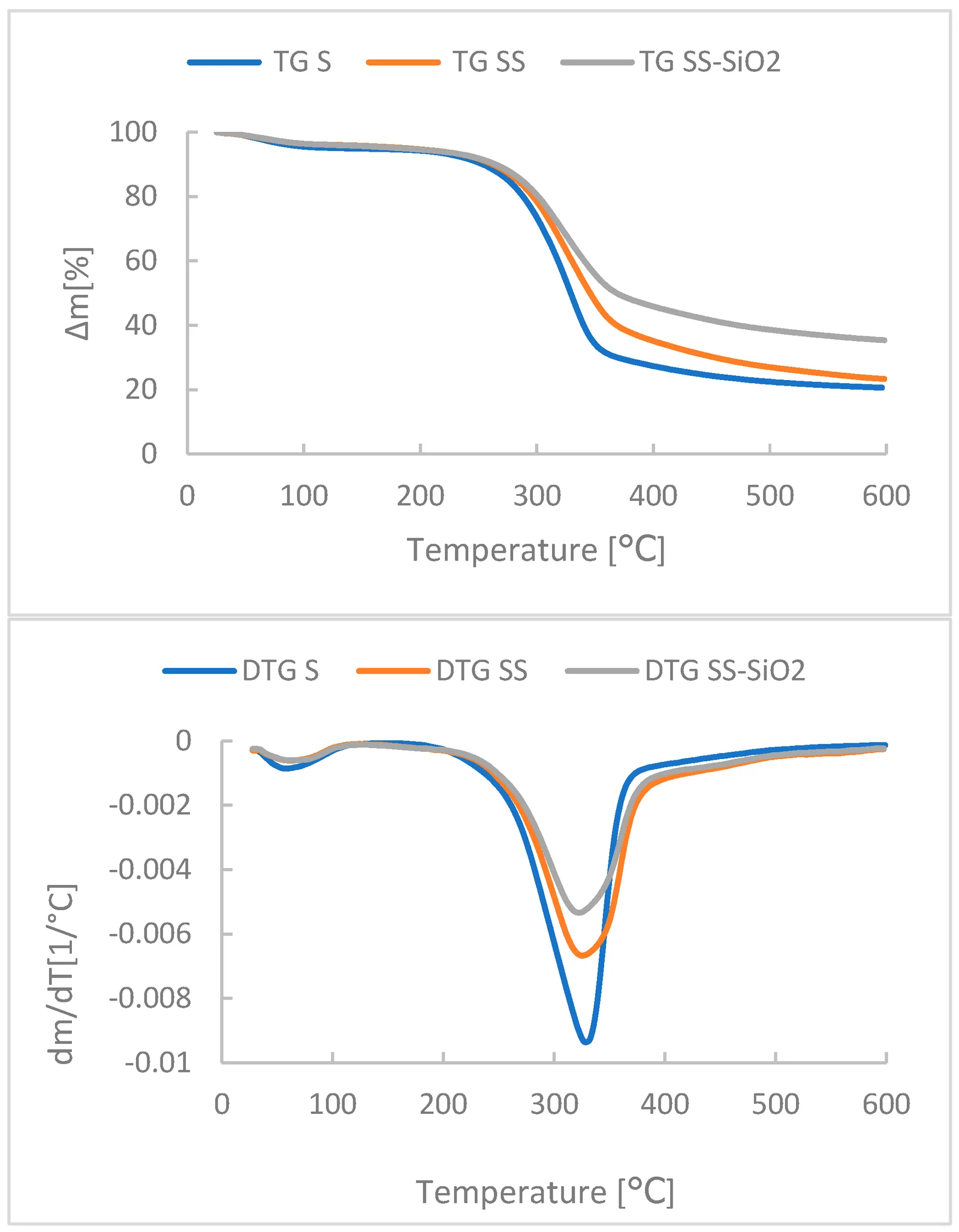
TG and DTG curves for pure and modified straw, as well as hybrid fillers.

Thermogravimetric analysis (TGA) is a valuable technique for assessing the thermal stability of materials by monitoring changes in sample mass as a function of temperature. Thermal stability refers to the material’s ability to maintain its characteristic properties within a defined temperature range, which is crucial for determining the optimal processing conditions of composites containing biofillers. In this study, the thermal degradation behavior of unmodified straw, chemically silanized straw, and a hybrid filler based on silanized straw modified with silica (Aerosil 380) was analyzed. The TG and DTG curves are presented in Figure 3, while the parameters determined based on these curves are summarized in Table 3.

Based on the curves, the thermal degradation of the materials can be divided into two distinct stages. The first stage occurred in the temperature range of 60–100 °C and corresponds to the evaporation of moisture physically bound to the lignocellulosic fibers. The weight loss in this range was approximately 6% for the unmodified straw and 4.5% for the silanized straw. The lower weight loss observed in the silanized sample indicates enhanced hydrophobicity of the material, resulting from the chemical modification of the fiber surface—hydroxyl groups responsible for water absorption were partially replaced by alkyl groups from the silane, thereby reducing the material’s hygroscopicity [28,29]. The second, main stage of thermal degradation is associated with the decomposition of lignocellulosic components—cellulose, hemicellulose, and lignin—and occurs over a broad temperature range (180–400 °C) [30]. In the case of unmodified straw, degradation started around 180 °C and reached its maximum rate at 330 °C. For both silanized straw and the hybrid filler, the onset of degradation shifted to approximately 200 °C, while the maximum decomposition rate (DTG peak) was observed at 340 °C. This shift reflects the improved thermal stability of the modified material. Silanization contributed to the partial removal of thermally less stable components such as hemicellulose and lignin and led to the formation of a protective surface layer that limits oxygen access and suppresses radical degradation mechanisms. Furthermore, the introduction of siloxane groups stabilized the fiber structure, enhancing its resistance to elevated temperatures [31].

The temperature at which 5% mass loss occurred (T_5_) further supports these findings: 127.0 °C for unmodified straw, 185.0 °C for silanized straw, and 181.7 °C for the hybrid filler. The increase in T_5_ in modified samples confirms improved resistance to the initiation of thermal decomposition. Total mass loss in the 25–600 °C range (ΔM_25–600_) also demonstrates the beneficial effects of modification. The unmodified straw exhibited a 79.4% mass loss, compared to 76.7% for silanized straw and only 64.6% for the hybrid filler. Reduced mass loss indicates slower degradation and greater thermal resistance. Additionally, the residual mass at 600 °C (R_600_) increased from 20.6% in unmodified straw to 23.3% in silanized straw and 35.4% in the hybrid filler. The higher residue in the hybrid filler is attributable to the thermal stability of the silica component (SiO_2_), which exhibits only minor mass loss within the studied temperature range, primarily due to the release of physically adsorbed water. In conclusion, both chemical silanization and further mechanical modification of straw fibers with silica significantly enhance the thermal stability of the material. These improvements result from changes in the chemical composition of the fibers and the structural stabilization induced by the incorporation of siloxane groups and thermally resistant silica. Such modifications render the obtained fillers more suitable for use in polymer composites processed at elevated temperatures, thereby broadening their application potential.

#### 3.1.5. Scanning Electron Microscopy (SEM) Analysis of Straw-Based Fillers

**Figure 4:**
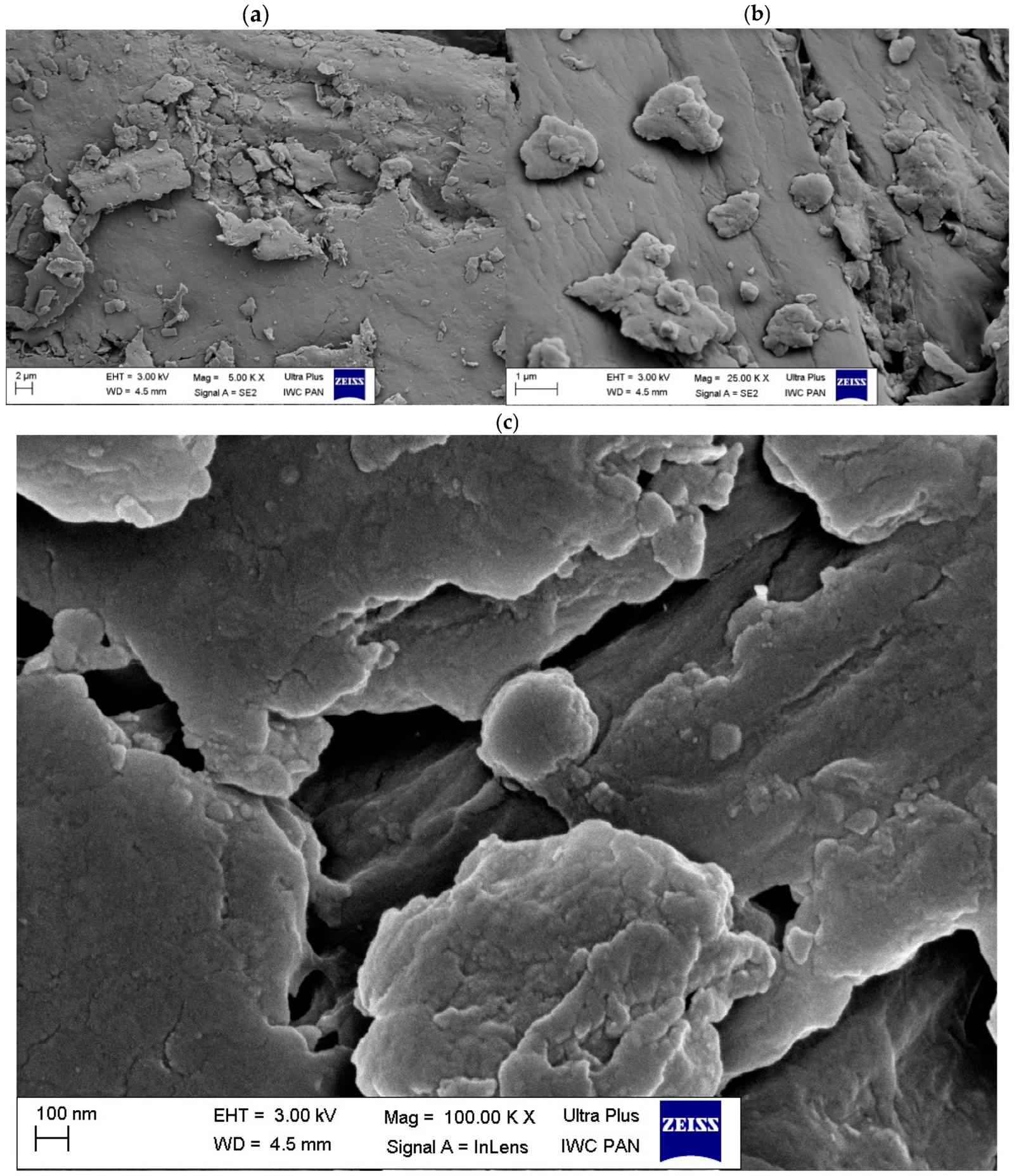
Microscopic images of straw fillers (S) at a magnification of (**a**) ×5.00 k, (**b**) ×25.000 k, (**c**) ×100.00 k.

**Figure 5:**
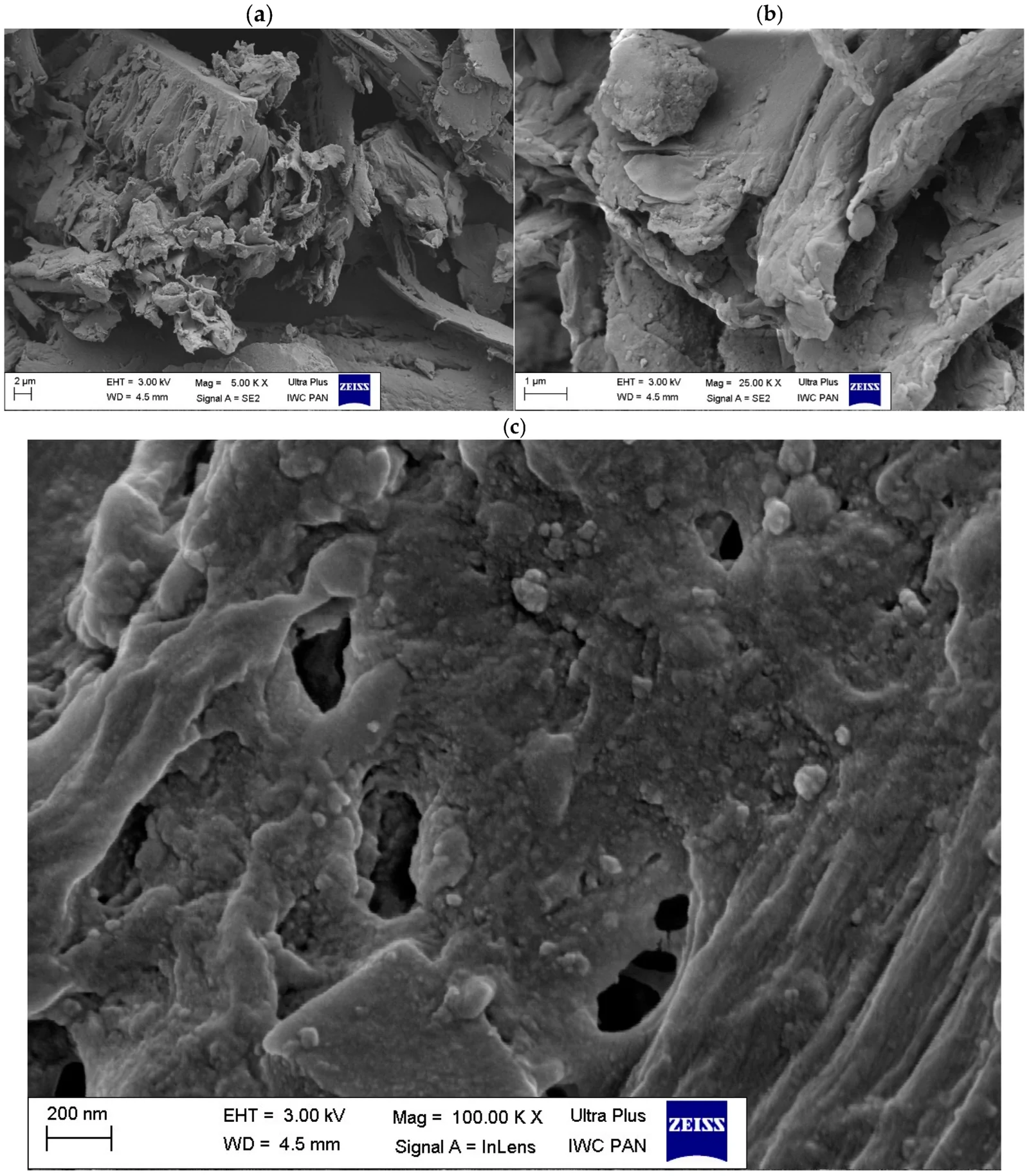
Microscopic images of silanized straw fillers (SS) at a magnification of (**a**) ×5.00 k, (**b**) ×25.000 k, (**c**) ×100.00 k.

**Figure 6:**
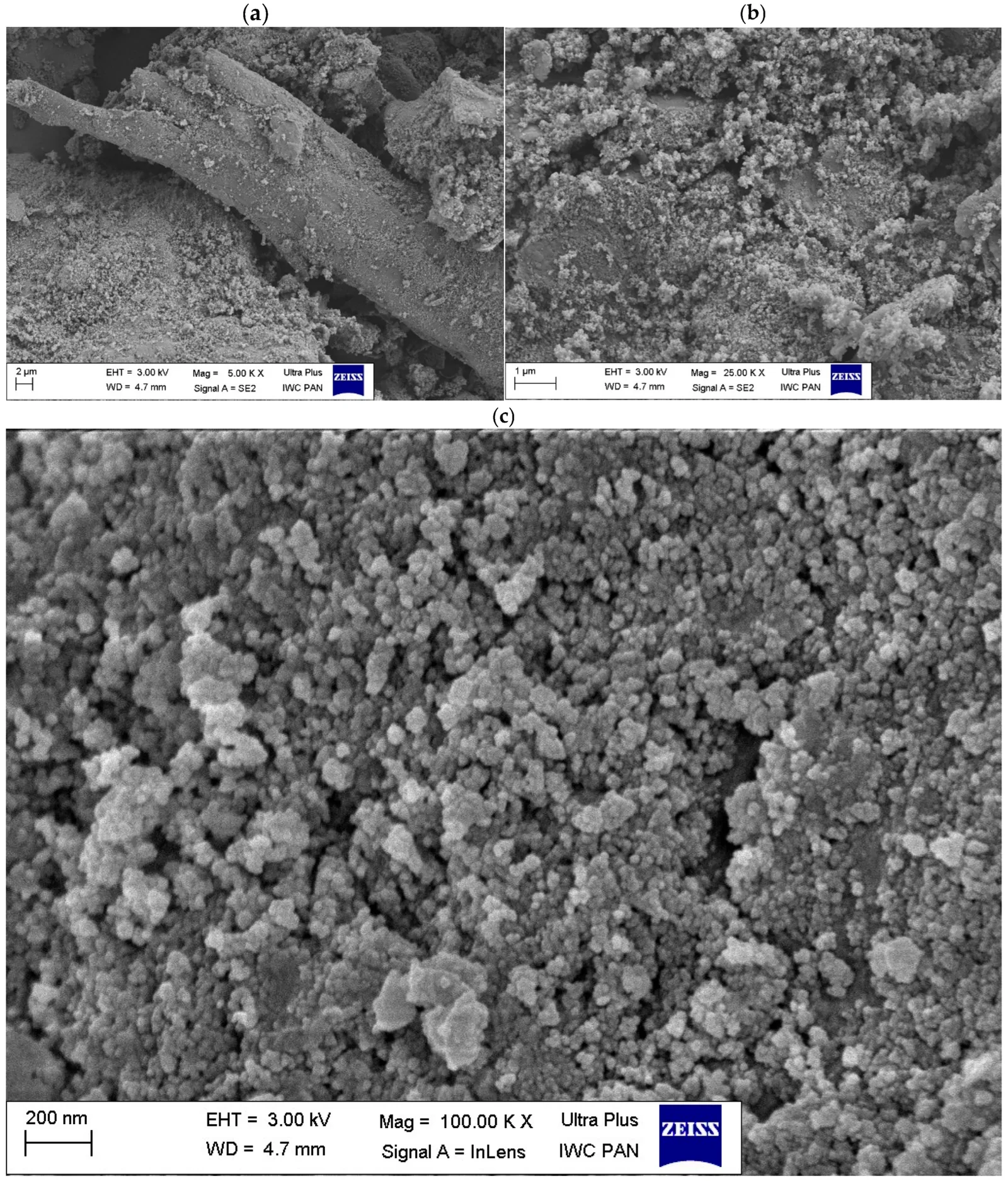
Microscopic images of hybrid straw fillers (SS-SiO_2_) at a magnification of (**a**) ×5.00 k, (**b**) ×25.000 k, (**c**) ×100.00 k.

Scanning electron microscopy analysis was performed on three types of straw-based fillers: unmodified (S), silanized (SS), and hybrid silanized straw-silica (SS-SiO_2_), to assess the impact of different modification methods on surface morphology. The SEM images were presented in Figure 4, Figure 5 and Figure 6, each recorded at three different magnifications: (a) ×5.00 k, (b) ×25.00 k, and (c) ×100.00 k. These correspond to successive enlargements (1:5 and 1:20) and allow multiscale observation of structural changes at progressively finer levels of detail.

In the case of unmodified straw fibers (Figure 4), the surface is characterized by high porosity, visible cracks, and an irregular, rough texture. Numerous fine particles and loosely attached impurities are evident. At higher magnifications (×25.00 k and ×100.00 k), a large number of surface defects become apparent, suggesting low cohesion and a high susceptibility to degradation. Such morphology may limit the effectiveness of these fibers as a reinforcing phase in composites, especially in terms of adhesion to a polymer matrix.

Silanization (Figure 5) significantly alters the fiber surface morphology. The structure becomes more compact, with a noticeable reduction in defects and porosity. A smoother surface and the formation of a thin protective layer are observed, likely resulting from condensation reactions between the silane and cellulose hydroxyl groups. Higher magnifications reveal fine spherical particles and localized indentations, though their distribution is more uniform than in unmodified fibers. This modification improves interfacial compatibility with the polymer phase, enhancing the material’s potential as a functional filler.

In contrast, the hybrid filler (Figure 6), which combines straw with nanosilica, exhibits a completely different surface morphology. Even at low magnification (×5.00 k), the straw is densely coated with a layer of fine, spherical silica particles. At higher magnifications, this coating appears as a continuous granular layer, significantly increasing both surface roughness and specific surface area. These features can enhance mechanical properties by improving interfacial adhesion and stress transfer within composite materials.

Raw straw (S) shows the highest porosity and irregularity, indicating limited application potential. Silanization (SS) significantly improves morphology by reducing surface defects and increasing homogeneity. The hybrid filler (SS-SiO_2_) demonstrates the most developed microstructure, combining the advantages of surface smoothing with the functional benefits of silica nanoparticles.

### 3.2. Foaming Behavior of Polyurethane Mixtures

**Figure 7:**
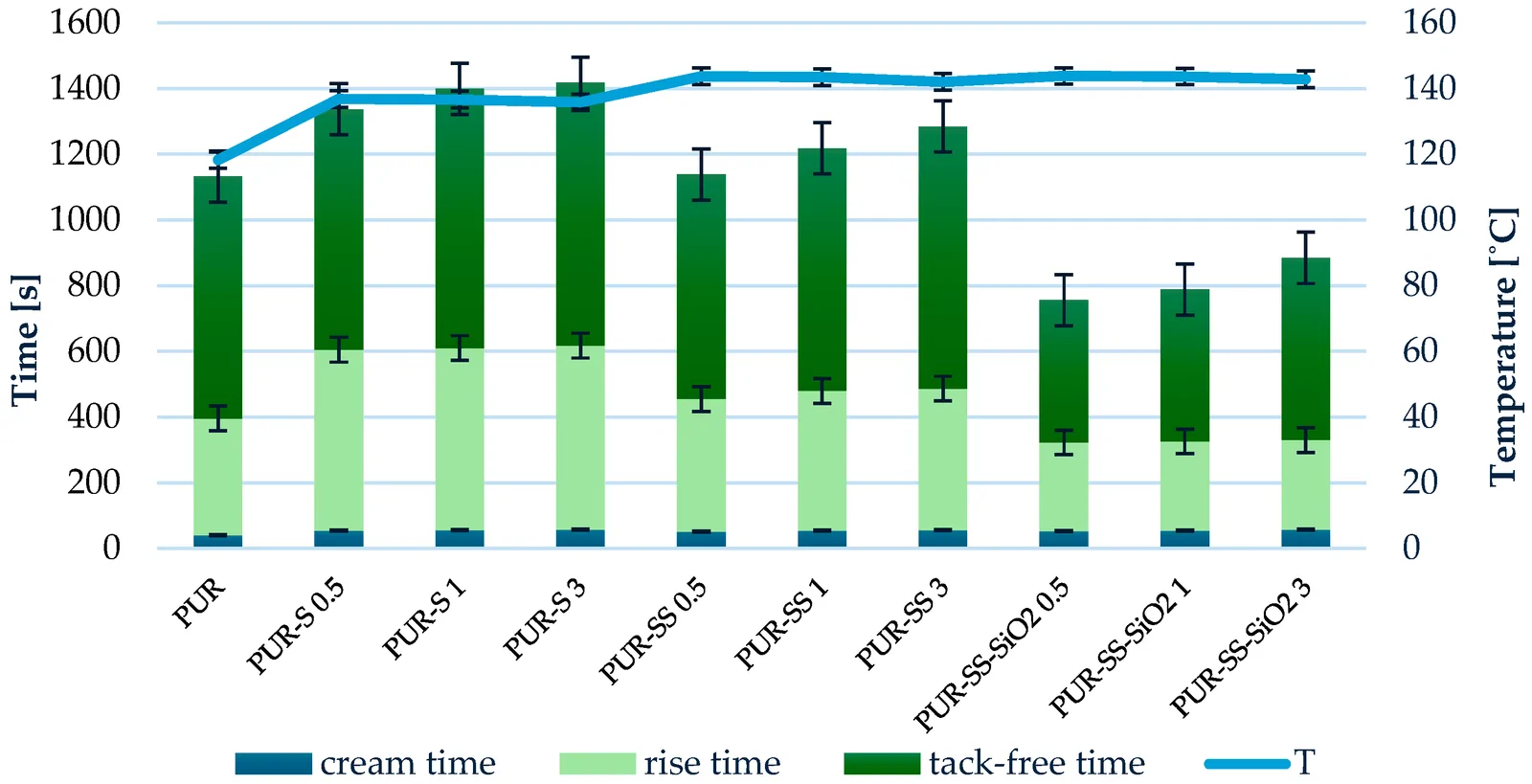
Dynamics of foam expansion taking into account latent time, growth, gelation (stabilization), and temperature increase obtained during PUR foams’ growth.

The foaming behavior was evaluated by monitoring the temperature profile and key processing times: cream time, rise time, and tack-free time. The synthesis of rigid polyurethane foams (PUR) is a highly exothermic process, during which a substantial amount of heat is released. The rate of temperature increase provides valuable insights into the reactivity of the polyol–isocyanate–additives system. The results of individual growth times and maximum synthesis temperature are presented in Figure 7.

Based on the presented chart, it is evident that, relative to the total synthesis time of the reference polyurethane foam, the incorporation of a filler in the form of unmodified or silanized straw resulted in a prolonged synthesis duration. In contrast, the introduction of silanized straw supplemented with silica contributed to a reduction in the overall reaction time. Furthermore, both the type and concentration of the filler significantly influenced the foaming kinetics. An increase in the filler content led to an extended cream time, rise time, and gel time. The prolonged cream time can be attributed to the presence of the filler in powdered form, which introduces additional nucleation sites, thereby delaying the onset of the foaming process. The extended rise time, in turn, was a consequence of increased system viscosity, which impeded mass transfer and slowed down the chemical reactions responsible for foam expansion. Although viscosity was not directly measured in the present work, our previous studies on polyol systems containing various organic and inorganic fillers consistently demonstrated a significant increase in dynamic viscosity with filler addition [32,33]. This effect provides a strong basis for interpreting the extended rise and gel times observed in the present systems.

The addition of fillers increased the maximum reaction temperature compared to the reference foam. This is due to the reaction of the hydroxyl-containing components (cellulose and silica) with isocyanates, which triggers secondary reactions that release additional CO_2_ and increase heat generation. The foams modified with silanized straw and silica showed the highest peak temperatures. However, increasing the filler content slightly reduced the maximum temperature, probably due to the higher viscosity of the system, which hindered foam expansion and partial heat absorption by the filler.

#### 3.2.1. Microstructure and Apparent Density of PUR Foams

Cell structure is one of the key factors determining the physical, mechanical, and functional properties of rigid polyurethane (PUR) foams. Its nature—including size, shape, arrangement, and the share of open and closed pores—directly affects the apparent density, thermal conductivity, mechanical strength, and dimensional stability of the finished material.

**Figure 8:**
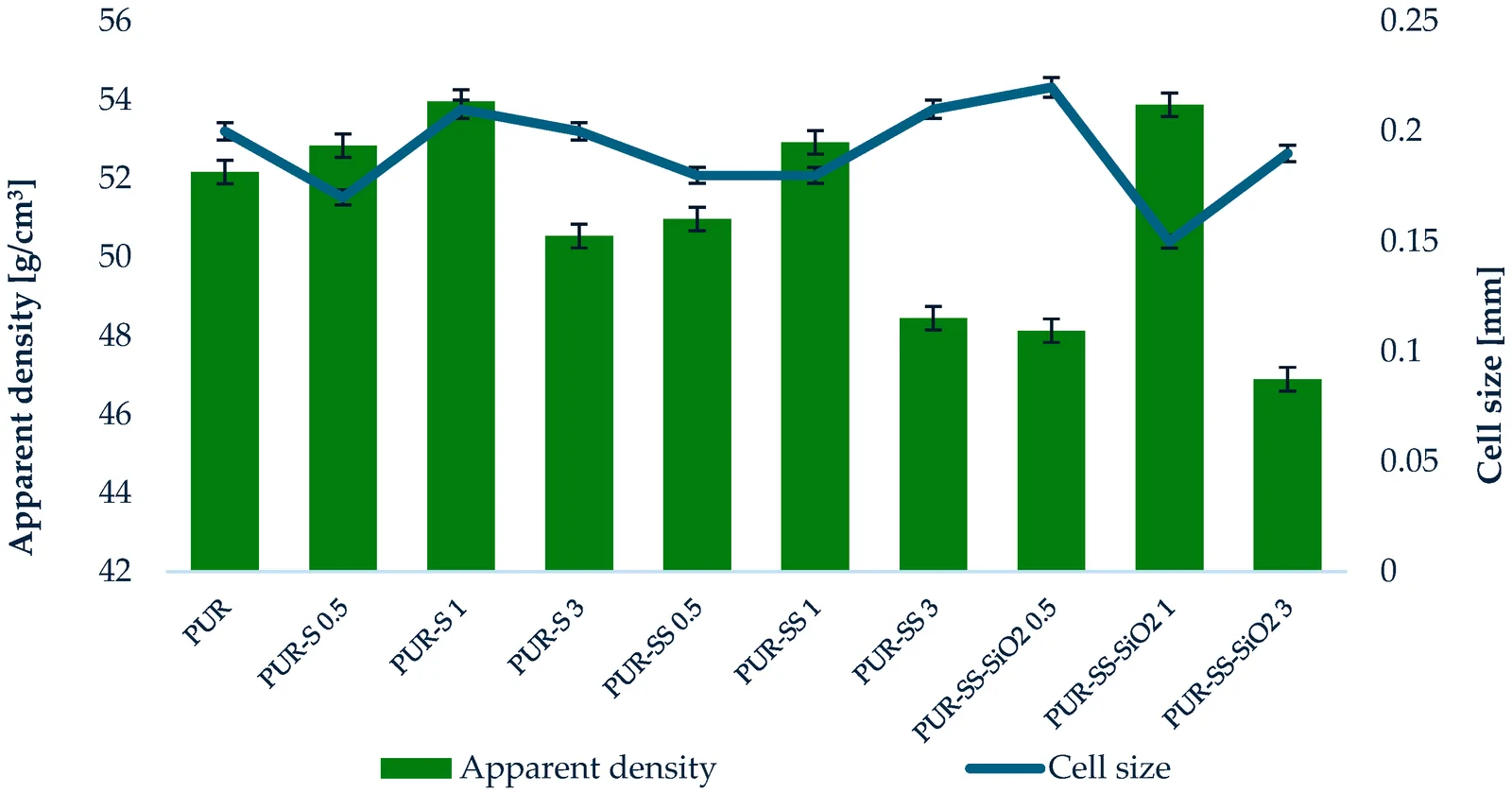
Pore size and apparent density of PUR foams.

The use of modifiers in the form of straw (unmodified, silanized, and silanized with added silica) significantly affected the structure of PUR foams (Figure 8). Increased porosity and the presence of larger, open pores are observed with the increase in filler content.

For foams containing unmodified straw, the addition of 0.5 parts by weight led to a decrease in the pore diameter compared to the reference foam, while 1 and 3 parts by weight increased the pore size and greater heterogeneity of the structure. A similar trend was observed in the case of silanized straw—the largest pores and irregular structure occurred with 3 parts by weight. Silanized straw with added silica had a different effect on the foams—already at 0.5 parts by weight, there was a significant increase in the pore diameter, but a further increase in the amount of filler led to a decrease in their size. Structures with 3 parts by weight were non-uniform, containing both small pores (resulting from high viscosity) and large, open pores (cell fusion effect).

Based on the analysis of the obtained apparent density results, it can be concluded that the addition of fillers in the form of straw—S, SS, and SS-SiO_2_—did not cause significant changes in the apparent density of the resulting rigid polyurethane foams compared to the unmodified systems. For each of the modified formulations, a slight initial increase in apparent density is observed at lower filler contents (0.5 and 1 parts by weight). This phenomenon results from the restricted expansion of the foam during the foaming process, caused by the increased viscosity of the reactive mixture, caused by the addition of fillers [33,34]. Higher viscosity hinders the free formation of pores, leading to a more compact structure with a higher proportion of closed cells and, consequently, higher apparent density.

In contrast, at a higher filler concentration (3 parts by weight), a decrease in apparent density is noted. In this case, the foam structure becomes looser and more porous, with a greater share of open cells. Despite the seemingly larger volume, this results in a reduction in the material’s apparent density.

#### 3.2.2. Mechanical Properties of PUR Foams

**Figure 9:**
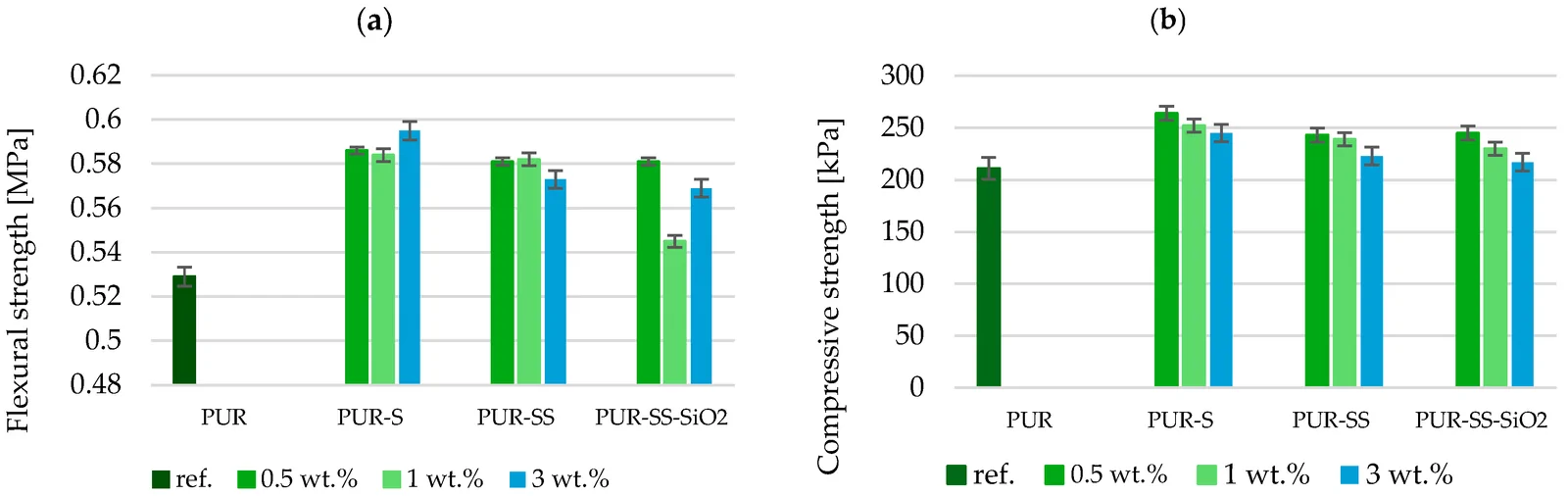
Mechanical properties of straw-modified PUR foams: (**a**) three-point bending, (**b**) compressive strength.

Based on the analysis of three-point flexural strength results presented in Figure 9, it can be observed that the addition of straw fillers led to an improvement in these mechanical properties. The increase in flexural strength indicates a beneficial effect of the applied modifiers on the internal structure of the foams, contributing to their reinforcement.

Three-point flexural strength is closely related to the foam’s pore morphology. The more porous the structure, especially with a higher proportion of open cells, the lower the stiffness and mechanical resistance. Therefore, at higher filler contents, where an increased presence of open cells is observed, a partial deterioration in mechanical performance occurs. Nevertheless, even at the highest filler concentrations, the flexural strength of modified foams remains higher than that of the reference (unmodified) foam, clearly confirming the positive impact of the additives used.

A similar trend can be observed in compressive strength measurements. The incorporation of straw—regardless of its chemical modification—positively affects the foam’s resistance to deformation under compressive loads. The most significant increase in compressive strength was noted at lower filler contents (0.5–1 parts by weight), which is associated with increased apparent density and a more compact cell structure dominated by closed cells.

As the filler content increases to 3 parts by weight, the foam structure becomes more open and porous, leading to a reduction in compressive strength. However, even in this case, the mechanical performance of the modified systems remains superior to that of the unmodified foam.

In conclusion, the use of straw as a filler—particularly after chemical modification—has a positive impact on both the flexural and compressive strength of rigid polyurethane foams. This demonstrates the potential of this biomass as an effective, environmentally friendly reinforcing additive in thermal insulation materials.

#### 3.2.3. Analysis of Water Absorption in PUR Foams

**Figure 10:**
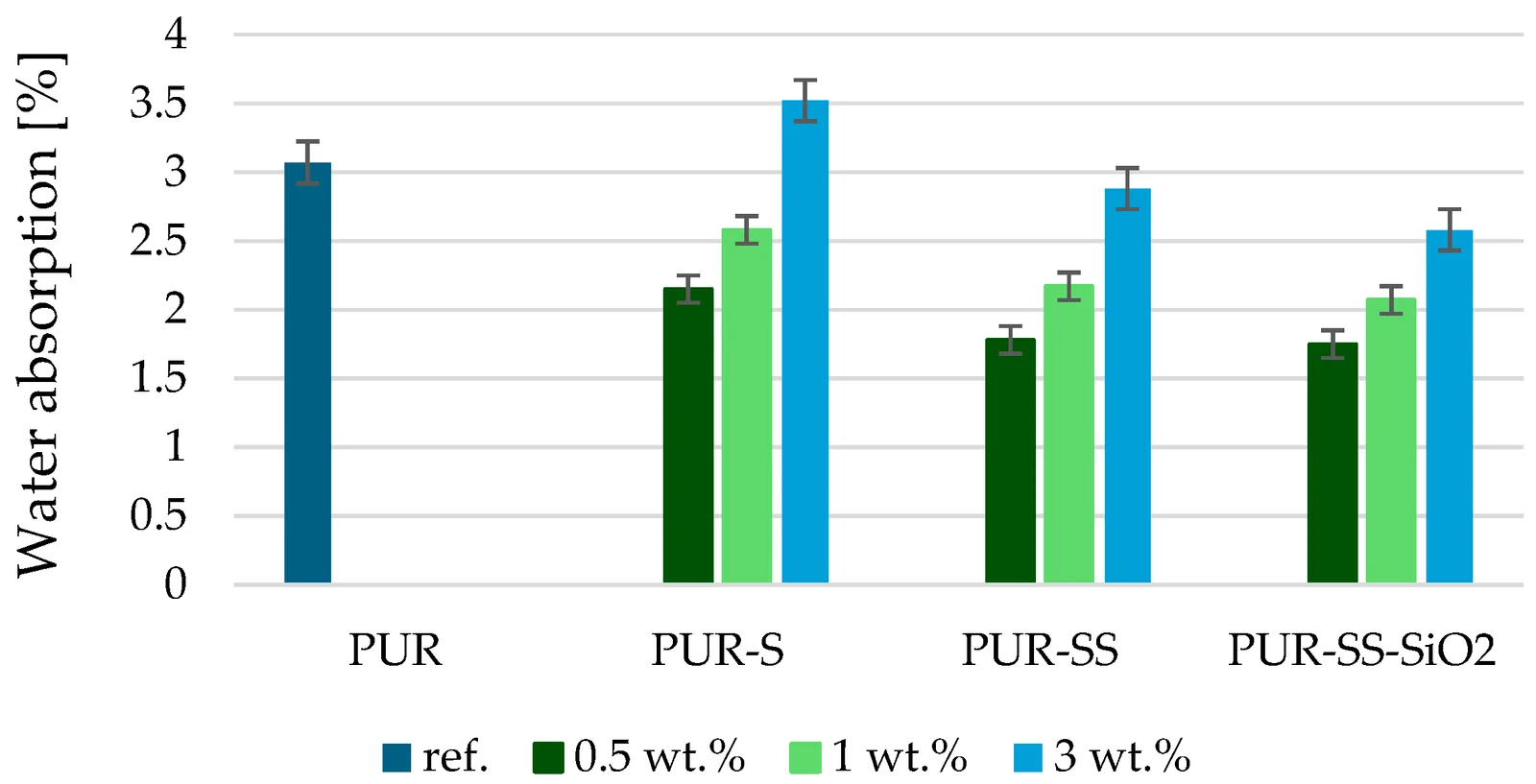
Short-term water absorption of rigid polyurethane foams (PUR) filled with straw.

Based on the results presented in Figure 10. It can be concluded that the applied modifiers had a noticeable effect on the water absorption properties of rigid polyurethane foams. The reference foam, which did not contain any fillers, exhibited the highest water uptake compared to the modified systems, indicating lower resistance to water absorption.

The incorporation of straw-based fillers—regardless of their modification method—led to a reduction in water absorption, thereby improving the water resistance of the foams. The best performance in this regard was observed for foams modified with silanized straw combined with silica, despite the inherently hydrophilic nature of silica. This effect can be attributed to improved filler–matrix interactions and a more favorable microstructure, which limits water penetration into the foam. The optimized water absorption resistance in polyurethane foams modified with silanized straw and fumed silica results from the synergistic effect of both modification processes. Silanization reduces the number of hydrophilic hydroxyl groups on the surface of cellulose fibers, reducing their natural tendency to absorb moisture. Simultaneously, it introduces functional groups capable of forming chemical bonds with the polyurethane matrix, improving adhesion at the interface and strengthening the material’s cohesion. The addition of pyrogenic silica acts as a high-surface-area nanofiller that stabilizes the fiber dispersion, prevents agglomeration, and increases surface roughness. This creates a more compact foam microstructure with a low filler content (0.5–1 wt.%), which limits water penetration.

The filler content was also a critical factor influencing water absorption. As the amount of filler increased, a rise in water absorption was observed. This trend is associated with the development of a more open-cell structure, where the higher proportion of open pores facilitates the ingress and retention of water within the foam material.

### 3.3. Dynamic Mechanical Analysis (DMA)

Dynamic Mechanical Analysis (DMA) was carried out to evaluate the thermomechanical properties of rigid polyurethane foams, including both the reference foam and those modified with straw in various forms: unmodified (PUR-S), silanized (PUR-SS), and silanized with the addition of silica (PUR-SS-SiO_2_). The analysis focused on the variation in storage modulus (G′) and loss modulus (G″) as a function of temperature at a frequency of 1 Hz. The results are presented in Table 4. The storage and loss modulus values (G′ and G″) represent the mean values of measurements on multiple pre-cut foam discs. For clarity, these values are presented as integers; the estimated standard deviation for these measurements is approximately ±1% of the mean value, reflecting both instrument precision and sample-to-sample variability.

The obtained analysis results enabled the determination of the temperature-dependent behavior of the storage modulus G′ and the loss modulus G″, particularly in the region of the glass transition. Based on the maximum values of both moduli presented in the table, it can be concluded that both the modified and reference foams exhibit a clear dominance of elastic over viscous properties (G′ > G″), which indicates that the materials undergo predominantly reversible deformations in response to applied mechanical forces.

For the reference foam (PUR), the value of the storage modulus G′ was 765,230 Pa, while the loss modulus G″ reached 177,364 Pa. The addition of unmodified straw (PUR-S) led to an increase in both moduli, especially at higher filler contents. For the foam containing 3 parts by weight of filler, G′ increased to 1,042,243 Pa and G″ to 224,684 Pa. Lower concentrations (0.5 and 1 part by weight) also showed a progressive rise in these values (G′: 804,891 and 992,845 Pa; G″: 195,905 and 205,832 Pa, respectively), confirming that the presence of plant fibers strengthens the mechanical structure, though it also results in more pronounced changes under elevated temperatures. A similar trend was observed in our previous research, in which ground cloves were used as a bio-filler in rigid polyurethane foams [34].

In the case of foams modified with silanized straw (PUR-SS), different trends were observed. The G′ value for the foam with 0.5 parts by weight of filler was 529,606 Pa—lower than that of the reference foam. However, at higher concentrations, the storage modulus increased significantly (977,725 and 1,063,710 Pa for 1 and 3 parts by weight, respectively). The loss modulus also increased with filler content—from 113,132 Pa (0.5 wt.%) to 200,690 Pa (3 wt.%). These results indicate that silanization improves the compatibility of the fibers with the polyurethane matrix, leading to better mechanical properties and more stable responses to mechanical loads at elevated temperatures.

Foams containing silanized straw with added silica (PUR-SS-SiO_2_) showed the lowest storage modulus at 0.5 parts by weight—only 227,319 Pa—and the lowest loss modulus at 66,699 Pa. However, with increasing filler content, the values steadily rose, reaching 559,174 Pa for G′ and 123,020 Pa for G″ at 3 wt.%. While these values were lower than in foams modified only with straw, they exhibited more stable, gradual changes under increasing temperatures.

The results obtained for the foam (PUR-SS-SiO_2_) demonstrate a compromise between the material’s stiffness and its stability at elevated temperatures.

This phenomenon may result from the synergy of chemical and physical modification. Silanization improves the compatibility of the fibers with the polyurethane matrix, reducing defects at the interface, while the presence of fumed silica stabilizes the filler dispersion and introduces additional mechanical bonds. This results in a more uniform microstructure, in which the local movements of the polymer segments are partially restricted, but not to the point of excessive stiffening. Thanks to this, the material does not show a sudden drop in modulus value during heating, but rather gradual and more controlled changes. In summary, the DMA results demonstrated that both the type and amount of filler significantly affect the mechanical and thermal properties of rigid polyurethane foams.

Modification with unmodified straw increases stiffness but also leads to sharper property degradation under heat. Silanization improves fiber–matrix interaction, particularly at higher filler contents. The addition of silanized straw combined with silica results in materials with lower modulus values but greater thermomechanical stability and the most gradual property changes within the glass transition range. Across all tested systems, elastic properties dominated over viscous ones (G′ > G″), confirming the foams’ ability to respond elastically and reversibly to applied stress.

## 4. Conclusions

This study aimed to obtain rigid polyurethane foams with improved performance properties by modifying their composition with cellulose in the form of straw—unmodified, silanized, and silanized—with the addition of silica, in amounts of 0.5; 1; and 3 parts by weight relative to the polyol mass. Both the type and amount of filler used had a significant impact on the synthesis process as well as on the physicochemical and mechanical properties of the resulting foams.

The results also allowed for the assessment of the fillers themselves. Unmodified straw, despite its natural origin and availability, has limited surface activity and lower thermal resistance. Silanization improves its thermal stability, reduces agglomeration, and increases compatibility with the polyurethane matrix. The addition of silica further increases the filler’s porosity and surface activity, contributing to enhanced mechanical and structural properties of the composites.

The presence of straw strongly influenced the foaming process. Unmodified and silanized straw extended the reaction time, whereas the addition of silica to silanized straw accelerated it. Structural analysis revealed that filler content controlled the porosity and cell structure of the foams. At 1 wt.% filler, the most favorable results were observed, with the highest apparent density and uniform morphology. By contrast, higher loading (3 wt.%) led to an increase in open-cell fraction and reduced structural homogeneity, which translated into a more porous but less compact structure.

Mechanical testing confirmed that the incorporation of straw can reinforce rigid polyurethane foams, but the effect was strongly dependent on filler concentration. The best compressive and flexural strength was achieved at 0.5 wt.% loading, where the balance between reinforcement and cell structure was optimal. Increasing the filler content beyond this point resulted in a gradual loss of strength due to excessive porosity and structural irregularities.

Dynamic Mechanical Analysis (DMA) confirmed that for all tested systems, elastic behavior (G′ > G″) dominated over viscous behavior, indicating the prevalence of reversible deformations. The introduction of fillers generally caused a reduction in both storage modulus G′ and loss modulus G″ with increasing content. Nevertheless, foams containing silanized straw with silica exhibited the most stable thermomechanical behavior, showing gradual and controlled changes in the glass transition region and thus better long-term resistance to elevated temperatures.

Water absorption tests highlighted another crucial advantage of filler modification. While all types of straw reduced water uptake compared to the reference sample, the most significant improvement was observed for silanized straw with silica, confirming the beneficial effect of combined chemical and inorganic modification. However, increasing filler concentration also increased the share of open cells, which in turn reduced water resistance at higher loadings.

In conclusion, the optimal balance of properties was achieved with a 0.5 wt.% filler content. Even a small amount of modified straw improved mechanical performance, thermomechanical stability, and reduced water absorption, while maintaining a favorable cellular structure. Considering its biodegradability, availability, and low cost, straw—especially after silanization and silica modification—represents an effective, sustainable, and eco-friendly biofiller for rigid polyurethane foam production.

## Figures and Tables

**Table 1:** Oil absorption number and maximum torque for the tested fillers.

Biofiller	DBPA [mL/100 g]	Max Torque [mNm]
S	109.5	191
SS	137.3	122
SS-SiO_2_	110.4	304

**Table 2:** Granulometric composition of the tested fillers, expressed as the percentage content of individual particle size fractions.

Biofiller	Sieve Fraction	Fraction Fn Content [%]
S	0.5–0.25	1.5 ± 0.1
	0.25–0.125	14.7 ± 0.3
	0.125–0.063	30.0 ± 0.4
	0.063–0.045	21.4 ± 0.3
	<0.045	29.9 ± 0.3
SS	0.5–0.25	4.6 ± 0.2
	0.25–0.125	14.3 ± 0.3
	0.125–0.063	23.9 ± 0.4
	0.063–0.045	24.4 ± 0.3
	<0.045	28.6 ± 0.3
SS-SiO_2_	0.5–0.25	3.2 ± 0.1
	0.25–0.125	17.3 ± 0.2
	0.125–0.063	20.6 ± 0.3
	0.063–0.045	19.7 ± 0.3
	<0.045	31.1 ± 0.4

**Table 3:** Summary of thermal analysis parameters (T_5_—initial decomposition temperature, (ΔM_25–600_)—mass loss between 25 °C and 600 °C, and R_600_—residue at 600 °C) determined from the TG and DTG curves for tested fillers.

Biofiller	T_5_ [°C]	ΔM_25__–__600_ [%]	R_600_ [%]
S	127.0	79.4	20.6
SS	185.0	76.7	23.3
SS-SiO_2_	181.7	64.6	35.4

**Table 4:** Dynamic Mechanical Analysis (DMA) determines the thermomechanical properties of rigid polyurethane foams (PUR) filled with straw.

	Storage Modulus G′ [Pa]			Loss Modulus G″ [Pa]		
PUR	765,230			177,364		
	0.5 wt.%	1 wt.%	3 wt.%	0.5 wt.%	1 wt.%	3 wt.%
PUR-S	804,891	992,845	1,042,243	195,905	205,832	224,684
PUR-SS	529,606	977,725	1,063,710	113,132	166,568	200,690
PUR-SS-SiO_2_	227,319	547,470	559,174	66,699	131,606	123,020

## Data Availability

The original contributions presented in this study are included in the article. Further inquiries can be directed to the corresponding author.
